# The Nephroprotective Effect of *Punica granatum* Peel Extract on LPS-Induced Acute Kidney Injury

**DOI:** 10.3390/life14101316

**Published:** 2024-10-16

**Authors:** Sena Sahin Aktura, Kazim Sahin, Levent Tumkaya, Tolga Mercantepe, Atilla Topcu, Esra Pinarbas, Zihni Acar Yazici

**Affiliations:** 1 Department of Medical Microbiology, Faculty of Medicine, Recep Tayyip Erdogan University, 53020 Rize, Turkeykazim.sahin@erdogan.edu.tr (K.S.); 2 Department of Histology and Embryology, Faculty of Medicine, Recep Tayyip Erdogan University, 53020 Rize, Turkey; 3 Department of Pharmacology, Faculty of Medicine, Recep Tayyip Erdogan University, 53020 Rize, Turkey; 4 Department of Biochemistry, Faculty of Medicine, Recep Tayyip Erdogan University, 53020 Rize, Turkey

**Keywords:** acute kidney injury, *Punica granatum*, sepsis, TLR4, NF-κB

## Abstract

Sepsis is an exaggerated immune response resulting from systemic inflammation, which can damage tissues and organs. Acute kidney injury has been detected in at least one-third of patients with sepsis. Sepsis-associated acute kidney injury increases the risk of a secondary infection. Rapid diagnosis and appropriate initiation of antibiotics can significantly reduce mortality and morbidity. However, microorganisms are known to develop resistance to antibiotics. Estimations indicate that the annual casualties caused by microbial resistance will surpass cancer fatalities by 2050. The prevalence of bacterial infections and their growing antibiotic resistance has brought immediate attention to the search for novel treatments. Plant-derived supplements contain numerous bioactive components with therapeutic potential against a variety of conditions, including infections. *Punica granatum* peel is rich in phenolic compounds. The purpose of this study was to determine the anti-inflammatory and anti-bacterial properties of *P. granatum* peel extract (PGPE) on lipopolysaccharide (LPS)-induced acute kidney injury. Experimental groups were Control, LPS (10 mg/kg LPS, intraperitoneally), PGPE100, and PGPE300 (100 and 300 mg/mL PGPE via oral gavage, respectively, for 7 days). According to biochemical results, serum blood urea nitrogen (BUN), creatinine (Cr) and C-reactive protein (CRP), kidney tissue thiobarbituric acid reactive substances (TBARS), and reduced glutathione (GSH) levels significantly decreased in the PGPE groups compared to the LPS group. Histopathological and immunohistochemical findings revealed that toll-like receptor 4 (TLR4) level and nuclear factor kappa B (NF-κB) expression increased in the LPS group compared to the Control group. In addition, the anti-Gram-negative activity showed a dose-dependent effect on *Acinetobacter baumannii*, *Escherichia coli*, and *Pseudomonas aeruginosa* with the agar well diffusion method and the minimal inhibitory concentration (MIC). The MIC value was remarkable, especially on *A. baumannii*. We conclude that PGPE has the potential to generate desirable anti-bacterial and anti-inflammatory effects on LPS-induced acute kidney injury in rats.

## 1. Introduction

Sepsis is a life-threatening condition that results from an irregular immune response caused by infection [1]. Organ dysfunction is one of the conclusions of sepsis that significantly affects the kidneys. Acute kidney injury has been detected in at least one-third of patients with sepsis [2]. Each year, more than 31 million people are diagnosed with sepsis, causing 5 million deaths globally [3]. Sepsis-associated acute kidney injury (S-AKI) increases mortality rates [2,4]. Newly developed renal dysfunction due to immune dysregulation during infection is a symptom of S-AKI. Creatinine levels and urine output are evaluated for the S-AKI diagnosis mentioned in the SOFA (Sequential Organ Failure Assessment) score used to assess organ dysfunction in the diagnosis of sepsis and in the KDIGO (Kidney Disease: Improving Global Outcomes) guideline criteria used to assess acute kidney injury [5]. In the SOFA score, acute kidney injury is diagnosed when the renal scoring creatinine level exceeds 1.2 mg/dL [5]; in the KDIGO guideline, the serum creatinine level increases by 0.3 mg/dL or 1.5–1.9 times or more than the baseline value [6].

Acute kidney injury often leads to immune dysfunction, increasing susceptibility to infections like urinary tract infections. AKI is associated with secondary infection [7]. The mortality rate caused by secondary infections is relatively high. In 57% of patients who developed acute kidney injury in the first week of admission to the intensive care unit, acute kidney injury was found to be due to sepsis [8]. Rapid diagnosis and appropriate antibiotic treatment can significantly reduce mortality and morbidity in S-AKI. Antibiotic resistance during therapy may result in higher mortalities [9]. Early initiation of antibiotics reduces the risk of developing acute kidney injury, and renal recovery is accelerated in patients with S-AKI [4]. Numerous microorganisms, including bacteria, viruses, fungi, and parasites, cause sepsis, but remarkably, the predominant causative microorganisms are Gram-negative bacteria [10]. Antibiotics inhibit the growth of bacteria or eliminate them, but inappropriate and widespread use of antibiotics causes antimicrobial resistance. Antimicrobial resistance refers to the ability of microorganisms to adapt and survive with genetic mutations [11]. The lack of awareness of antibiotic resistance in society has led to global problems. Estimations indicate that the annual casualties caused by resistant microorganisms exceed 10 million and will surpass cancer fatalities by 2050 [12]. The increasing prevalence of bacterial infections and their growing resistance to multiple drugs have led to searching for alternative treatments, including plants and plant-derived supplements [13]. The use of herbal medicine for treatment goes back to 3000 BC [13]. Traditional medicine initiated by indigenous cultures continues to be relevant, and 80% of the world’s population still uses herbal medicines for primary health care [14]. Compounds such as flavonoids, phenolics, alkaloids, terpenoids, tannins, and essential oils are the basis for antibiotic development [15]. The antibacterial and antifungal properties of crude extracts have been investigated [16].

Extensively grown worldwide as a fruit, *Punica granatum* (pomegranate) originates from the central Asian regions [17]. Since ancient times, *P. granatum* has served as a traditional medicine with numerous pharmacological properties, including antioxidant, antidiabetic, antidiarrheal, anticancer, antibacterial, antifungal, and anti-inflammatory [17,18,19,20,21,22,23]. The major pharmacological properties of *P. granatum* are derived from having a high content of polyphenols [23,24]. *P. granatum* peel is a phenolic compound resource that inhibits free radicals and shows excellent antioxidant properties [25,26]. Polyphenols scavenge free radicals and inhibit lipid oxidation in vitro [27]. Reduced glutathione (GSH) is a significant antioxidant in the body, playing a critical role in protecting cells from oxidative damage by scavenging free radicals and reactive oxygen species (ROS) [28]. Higher levels of GSH typically correlate with increased antioxidant capacity, as GSH directly neutralizes ROS [29]. In sepsis, the body is typically under increased oxidative stress, leading to higher production of free radicals that can cause cellular damage. In response, the body often produces more antioxidants, such as GSH. Elevation of GSH levels serves as a protective mechanism against cellular damage and contributes to the defense against oxidative stress [30]. The other important parameter in showing the level of damage occurring in issues under the effect of ROS is thiobarbituric acid reactive substances (TBARS). TBARS and malondialdehyde (MDA) are compounds that arise as a result of lipid peroxidation in cells. MDA is one of the most important final products of lipid peroxidation, and the TBARS test is typically used to determine MDA concentration [31]. Lipopolysaccharide (LPS, endotoxin) can lead to oxidative stress and lipid peroxidation, subsequently increasing TBARS levels [32]. Increased TBARS levels have been shown in response to LPS-induced inflammation.

Sepsis is a potential outcome that can result from excessive and uncontrolled pro-inflammatory signaling triggered by toll-like receptor 4 (TLR4) [33]. TLR4 is a toll-like receptor family member and representative of pattern recognition receptors. TLR4 is expressed in immune cells, mainly of myeloid origin, as well as non-immune cells, like endothelial cells [34]. TLR4 is activated by a significant component of the outer membrane of Gram-negative bacteria, LPS, and triggers pro-inflammatory reactions facilitating eradication of the invading bacteria [33]. The binding of TLR4 to acyl chains and phosphate groups of lipid A of LPS induces pro-inflammatory responses [33]. As a result of a series of signal activations, the nuclear factor kappa B (NF-κB) transcription factor expression is increased [35]. TLR4/NF-κB is a crucial inflammatory signaling transduction pathway that is associated with the pro-inflammatory response, cell differentiation, proliferation, and apoptosis [36]. The NF-κB signaling pathway induces the expression of genes encoding pro-inflammatory mediators, such as tumor necrosis factor α (TNF-α) [35]. Understanding the TLR4/NF-κB signaling transduction pathway is crucial for diagnosing and treating sepsis and S-AKI [33,34,35,36].

## 2. Materials and Methods

### 2.1. The Procurement and Extraction of P. granatum

*P. granatum* was bought from a public bazaar (Adiyaman, Turkey). The pomegranate was washed and peeled. The peels were dried at 40 °C and then ground into powder. Then, 15–20 g powder was extracted in a methanol-water mixture at 80:20 for 72 h. The extract was filtered through a Whatman paper, and the filtrates were concentrated using rotary evaporation (LabTech.EV311). *P. granatum* peel extract (PGPE) was dissolved in distilled water.

### 2.2. HPLC Analysis

The composition of PGPE used in the study was determined by high performance liquid chromatography (HPLC) (Section 3.1). The process followed the protocol described by Seyis et al. [37].

### 2.3. Animal Experiment and Drug Administration

The study involved 36 male Sprague–Dawley rats weighing 300 ± 20 g. The rats were kept under controlled light conditions (12 h light/12 h dark), 55–60% humidity, and a temperature of 22 ± 3 °C. They had access to food and water ad libitum. The rats were randomly divided into four groups: Control (orally isotonic saline treated), LPS (sepsis), and treatment groups PGPE100 and PGPE300. In the treatment groups, 100 and 300 mg/mL PGPE extracts were administered via oral gavage for 7 days, respectively. Additionally, 10 mg/kg LPS (Lipopolysaccharides from *E. coli* O55:B5 Sigma-Aldrich, Cambridge, MA, USA) was intraperitoneally (i.p) administered to all animals except those in the Control group. No procedure was performed on the animals in the Control group. The rats were anesthetized with ketamine-xylazine after 16–20 h of LPS injection. Blood and kidney tissue samples were collected for biochemical and histopathological analysis.

### 2.4. Biochemical Analysis

#### 2.4.1. Determination of Serum Blood Urea Nitrogen (BUN), Creatinine (Cr) and C-Reactive Protein (CRP) Levels

Serum BUN, Cr, and CRP levels were measured with particle-enhanced immunonephelometry using an image analyzer (Beckman Coulter, AU680, Brea, CA, USA).

#### 2.4.2. Determination of Tissue Thiobarbituric Acid Reactive Substances (TBARS) and Reduced Glutathione (GSH) Levels

The kidney homogenates were used for measurements. The TBARS levels were determined according to the method described by Ohkawa et al., and the results were expressed as nmol/g tissue [38].

The GSH levels were measured according to the Ellman method and the results were expressed as µmol/g tissue [39].

### 2.5. Histopathological Analysis

The kidney tissues were trimmed to a 1.5 cm^3^ volume and fixed in a 10% phosphate-buffered formalin (Sigma Aldrich, Darmstadt, Germany) solution for 36 h. The tissue samples were subjected to routine tissue preparation procedures using a tissue processor (Citadel 2000, ThermoScientefic, Waltham, MA, USA). The samples were taken through increasing ethanol concentrations (Merck GmbH, Darmstadt, Germany) for dehydration, followed by mordanting in xylol solution (Merck GmbH, Germany). They were then embedded in soft paraffin (Merck GmbH, Darmstadt, Germany) for 1 h and then blocked in hard paraffin (Merck GmbH, Darmstadt, Germany) overnight. Sections of 4–45 μm thickness were obtained with a rotary microtome (Leica RM2525, Leica Biosystems, Wetzlar, Germany). The sections were lined with Harris hematoxylin and Eosin G (H&E; Merck, GmbH, Darmstadt, Germany).

### 2.6. Immunohistochemical (IHC) Analysis

The inflammation pathway was demonstrated using the anti-Toll-like Receptor 4 (TLR4; Rabbit polyclonal antibody, a14637, Antibodies.com, Cambridge, UK) and the Nuclear Factor Kappa B (NF-κB/p65; Rabbit polyclonal antibody, ab16502, Abcam, Cambridge, UK) primary antibodies along with an appropriate secondary antibody (Goat Anti-Rabbit IgG H&L (HRP), ab205718, Abcam, UK).

Kidney sections were placed onto positively charged slides (Patolab, PRC). The sections underwent dehydration, 30% H_2_O_2_ treatment, following the instructions of the primary antibody kit manufacturer. TLR4 and NF-κB primary antibodies were incubated with the sections for 1 h, followed by incubation with secondary antibodies for another hour. Counterstaining was applied using diaminobenzidine tetrahydrochloride (DAB, Sigma Chemical, St. Louis, MO, USA) and Harris hematoxylin (Merck GmbH, Darmstadt, Germany).

### 2.7. Semi-Quantitative Analysis

A semi-quantitative analysis was calculated using the tubular necrosis scoring (TNS) of Sung et al. [40]. Tissue sections stained with H&E were graded as shown in Table 1 and Table 2. A total of 25 different randomly selected areas on each section were evaluated by two histopathologists blinded to the study groups.

### 2.8. Antibacterial Assay

#### 2.8.1. Agar Well Diffusion Method

The agar-well diffusion method was used to determine the antimicrobial effect of PGPE against *Acinetobacter baumannii*, *Escherichia coli*, and *Pseudomonas aeruginosa* following the procedure of Onaran Acar et al.’s [41] at 200 mg/mL PGPE. Bacterial strains used in the study were the isolates obtained from clinical samples at Recep Tayyip Erdogan University (RTEU) Education and Research Hospital. The sensitivity of different bacterial strains to PGPE was calculated by measuring the diameter (in millimeters) of the inhibition zone.

Antibiotics and pyrogen-free sterile water were used as positive and negative controls, respectively. The sensitivity pattern of the reference strains of bacteria was compared with the six commonly employed antibiotics: Amikacin (AMK), Ampicillin (AMP), Ceftazidime (CAZ), Ciprofloxacin (CIP), Meropenem (MER), and Piperacillin/Tazobactam (TZP).

#### 2.8.2. MIC (Minimum Inhibitory Concentration) Assay

The minimum inhibitory concentration was determined by the Clinical and Laboratory Standards Institute (CLSI) guidelines [42]. The standard broth dilution sensitivity method was employed to determine the MIC of bacteria to PGPE [43,44]. The experimental groups were PGPE-treated, negative control, and blank. The PGPE was diluted from 256 µg/mL to 2 µg/mL using Mueller–Hinton broth (MHB) medium. The MIC was determined by observing the clarification of the wells visually. The clarified well with the lowest concentration was identified as the MIC value.

### 2.9. Statistical Tests

Histopathological, immunohistochemical and biochemical data were analyzed using SPSS 18.0 statistical software (IBM Corp. Chicago, IL, USA) with the Shapiro-Wilk test, Q-Q plot, Skewness-Kurtoisis and Levene tests for their compliance with normal distribution. The parametric data obtained from biochemical analyses were calculated as mean ± standard deviation and differences between groups were subjected to one-way ANOVA followed by a Tukey HSD test. The nonparametric data obtained as a result of histochemical and immunohistochemical analyses were calculated as median and 25% and 75% interquartile ranges, and genetic transitions between groups were evaluated with Kruskal Wallis and Dunn tests. A value of *p* ≤ 0.05 was accepted as permanently significant.

## 3. Results

### 3.1. HPLC Analysis

The *P. granatum* peel extracts were analyzed by using HPLC to obtain phenolic compounds. The amounts were calculated in µg/g equivalent value to their HPLC spectrums and given in Table 3. The richest phenolic compound was punicalagin (PUN), observed at 50% MeOH extraction.

### 3.2. Biochemical Results

#### 3.2.1. BUN, Cr and CRP Levels

**Figure 1:**
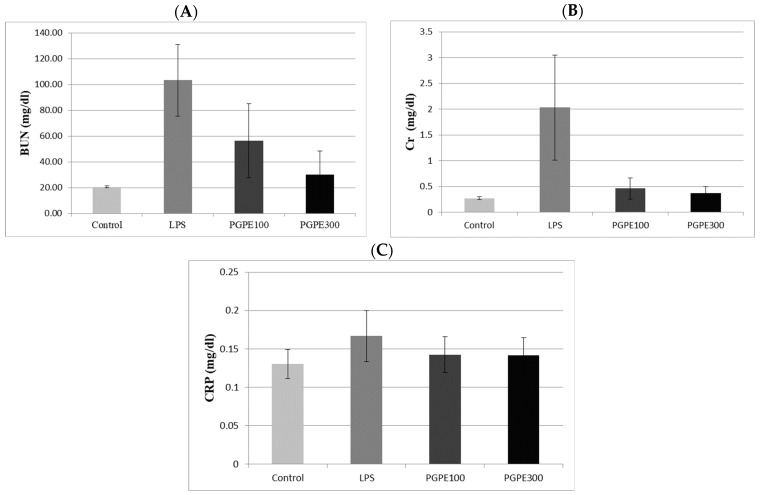
Serum blood urea nitrogen; BUN (**A**), creatinine; Cr (**B**), and C-reactive protein; CRP (**C**) Levels.

Renal function tests, including BUN and Cr levels, were used to analyze the damage in sepsis-associated acute kidney injury. In addition, CRP levels were evaluated to detect the severity of the inflammation. The results showed that all biomarker levels were significantly increased in the LPS group compared with the Control group (*p* < 0.05). In addition, the PGPE300 group experienced a more intense decretion of the levels than the PGPE100 group. The difference between the PGPE100, PGPE300, and LPS groups was statistically significant (*p* < 0.05) (Figure 1).

#### 3.2.2. TBARS and GSH Levels

The TBARS level in the Control group (115 ± 13 nmol/g tissue) increased to 223 ± 51 in the LPS group. However, PGPE treatment groups reduced TBARS levels to 147 ± 35 and 146 ± 17, respectively, as shown in Table 4. The difference between the LPS and Control group was statistically significant (*p* ≤ 0.001). No significant differences were found between the PGPE100, PGPE300, and Control groups (*p* > 0.05).

GSH level in the Control group (7.93 ± 0.85 μmol/g tissue) increased to 10.63 ± 2.47 μmol/g tissue in the LPS group. In the PGPE100 and PGPE300 groups, the GSH levels were 8.70 ± 1.04 and 8.85 ± 1.69 μmol/g tissue, respectively. The GSH levels revealed a statistically significant difference between the LPS group and the Control group (*p* < 0.05). No statistically significant differences were observed between the PGPE100, PGPE300, and Control groups (*p* > 0.05). The results are shown in Table 4.

### 3.3. Histopathological Analysis

**Figure 2:**
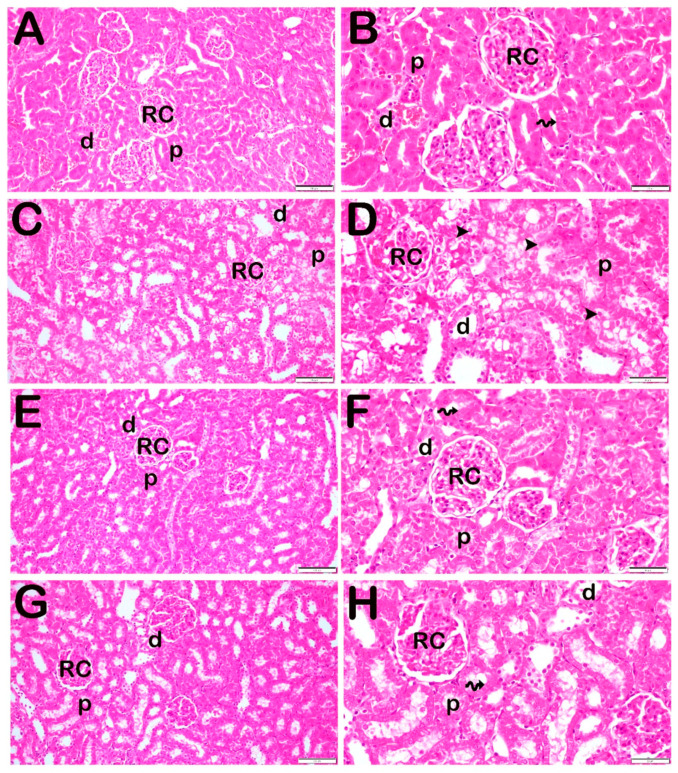
Representative light microscopy image of kidney tissue sections. Sections stained with H&E. Distal tubules (d), Proximal tubules (p), Renal corpuscle (RC). The brush border (spiral arrow). (**A**) (×20), (**B**) (×40), Sections from the Control group exhibiting normal renal tubules (p, d) and renal corpuscle structure (RC). The brush border (spiral arrow) is particularly evident in the proximal (p) tubules, and the distal (d) tubules are observed to be normally structured. [TNS: 0 (0–0.5)]. (**C**) (×20), (**D**) (×40)**:** Vacuolization in the renal epithelial cells and loss of brush border (arrowhead) structures was observed in the proximal epithelial cells in the LPS group. In addition, debris accumulation is observed in the renal tubules [TNS: 7.5 (5–8)]. (**E**) (×20), (**F**) (×40) Sections of PGPE100 treatment group exhibit a decrement in the loss of epithelial cells in the kidney tubules [TNS: 1.5 (0.5–2)]. (**G**) (×20), (**H**) (×40) In addition to exhibiting a decrement in the loss of epithelial cells in the kidney tubules, widespread tubule epithelial cells with typical epithelial appearance are observed [TNS: 1 (0.5–1)].

H&E-stained sections were examined under light microscopy. The Control group revealed normal renal corpuscles and proximal and distal tubule structures. In addition, the renal corpuscle, which consists of the glomerulus and the Bowman’s capsule, existed. (Figure 2A,B, Table 5; TNS: 0 (0–0.5)). On the contrary, widespread vacuoles in the cytoplasm of the degenerative renal corpuscle and renal tubular epithelial cells and accompanying necrotic tubules were observed in the LPS group. Accumulations of tubular luminal debris were observed therewith. In particular, the loss of brush border structures in proximal tubule epithelial cells was remarkable (Figure 2C,D, Table 5; TNS: 7.5 (5–8)). In contrast, in the PGPE100 group, a decrease in vacuolization of the proximal and distal tubules, accompanied by necrotic epithelial cells and debris accumulation in the renal tubules was evident. Brush structures were typical in proximal tubule epithelial cells (Figure 2E,F; Table 5; TNS: 1.5 (0.5–2)). Similarly, in the PGPE300 group, we observed decreased necrotic epithelial cells in the proximal and distal tubules and debris accumulation in the renal tubules. In addition, epithelial cells in the proximal tubules had a typical structure (Figure 2G,H; Table 5; TNS: 1 (0.5–1)).

### 3.4. IHC Analysis

#### 3.4.1. TLR4 Positivity

**Figure 3:**
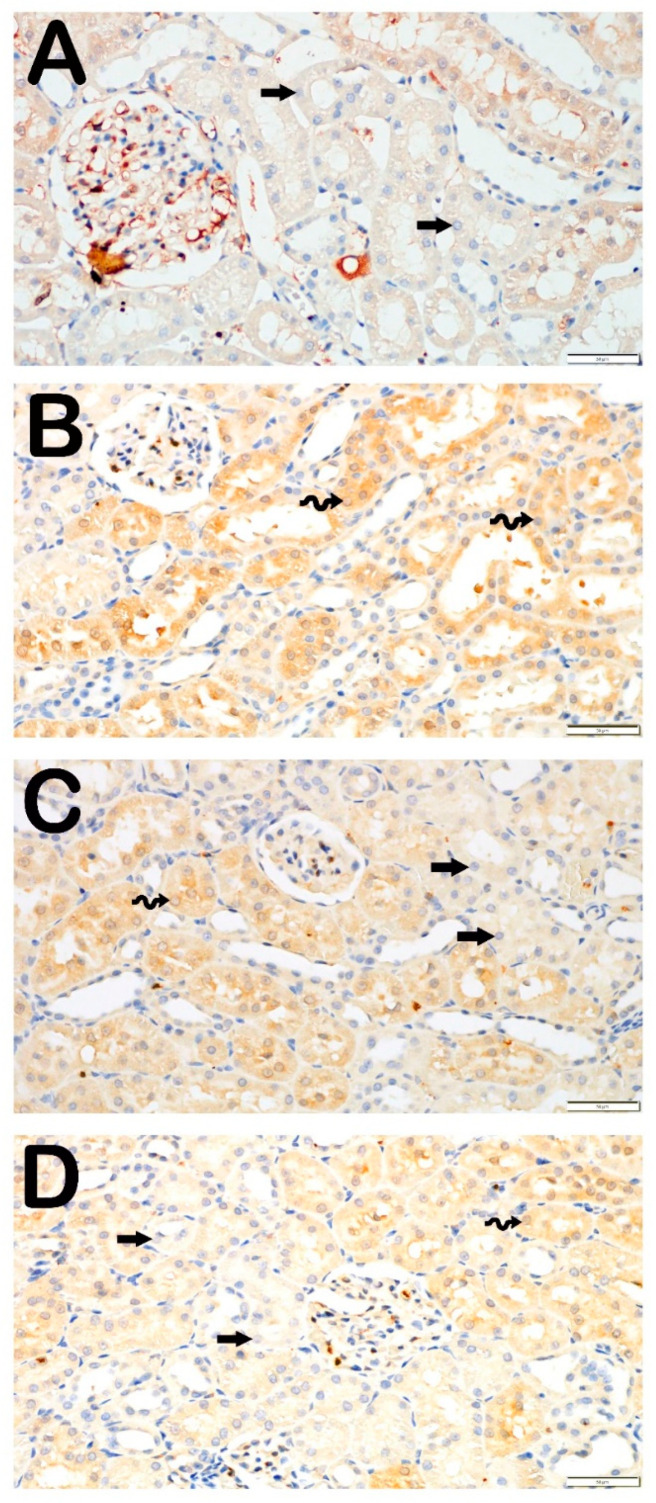
Representative light microscopy image of the effect of PGPE treatment on inflammatory (TLR4) changes after LPS-induced kidney injury. Typical tubule epithelium (arrow). (**A**) (×40)**,** Control group renal tubule epithelium (arrow) was observed to have a normal structure in the renal cortex [TRL4 positivity score: 0 (0–0.5)]. (**B**) (×40), LPS group sections demonstrated many apoptotic renal epithelial cells (spiral arrow) showed intensive TRL-4 positivity [TRL4 positivity score: 1.5 (1–2)]. (**C**) (×40)**,** PGPE100 group sections showed decreased apoptotic cells (spiral arrow) in renal tubule epithelium that showed TRL-4 positivity [TRL4 positivity score: 0.5 (0–0.5)]. (**D**) (×40)**,** A decreased TLR4 positive cell in the apoptotic (spiral arrow) renal tubule epithelium and typical epithelial cells (arrow) were observed widely in the PGPE300 group sections [TRL4 positivity score: 0.5 (0–0.5)].

Examination under light microscopy of kidney tissue sections incubated with TLR4 primary antibodies revealed a higher TLR4 positivity score in the LPS group (1.5 (1–2)) than in the Control group (0 (0–0.5)) (Figure 3A,B; Table 6). However, the TLR4 positivity score of 1.5 (1–2) in the LPS group decreased to 0.5 (0–0.5) in the both of PGPE100 and PGPE300 groups (Figure 3C,D; Table 6).

#### 3.4.2. NF-κB/p65 Positivity

**Figure 4:**
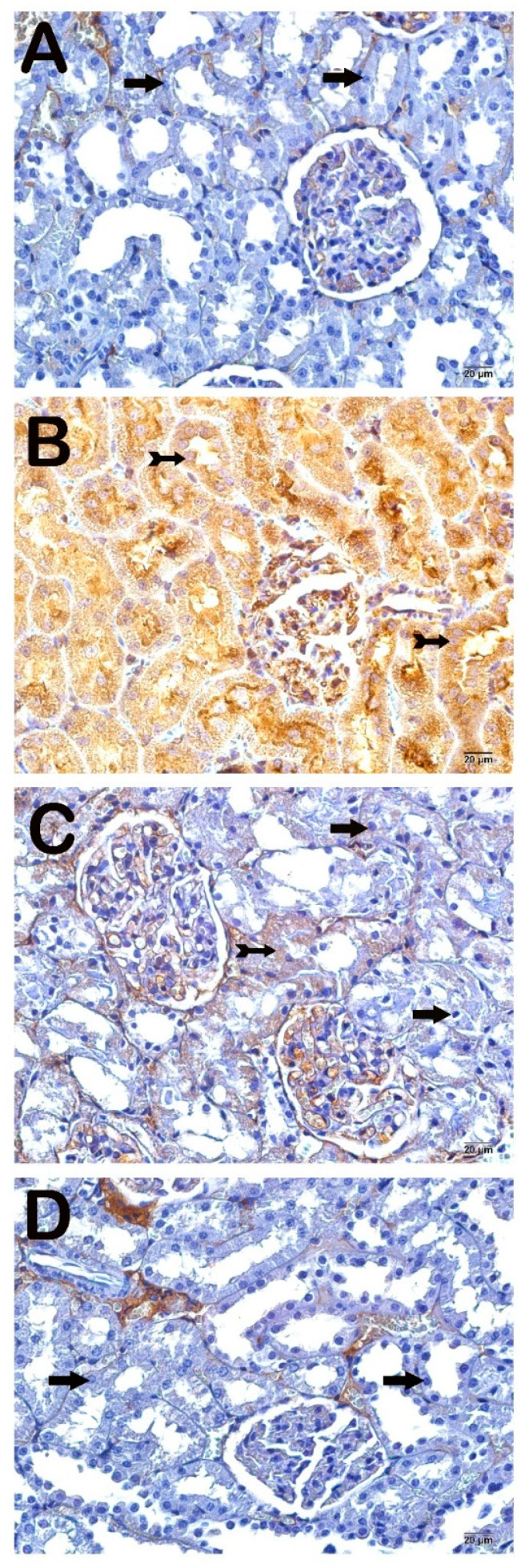
Representative light microscopy image of the effect of PGPE treatment on inflammatory (NF-κB/p65) changes after LPS-induced kidney injury. Typical tubule epithelium (arrow). (**A**) (×40), Control group kidney sections exhibit renal tubule epithelium (arrow) with a normal structure in the renal cortex [NF-κB/p65 positivity score: 0 (0–0)]. (**B**) (×40), LPS group sections demonstrated many apoptotic renal epithelial cells (tailed arrow) showed intensive NF-κB/p65 positivity [NF-κB/p65 positivity score: 3 (2–3)]. (**C**) (×40), PGPE100 group sections showed decreased NF-κB/p65 positive cells (tailed arrow) in the kidney cortex [NF-κB/p65 positivity score: 0 (0–1)]. (**D**) (×40), PGPE300 group sections showed decreased immune-positive cells in the renal tubule epithelial cells, and widespread typical epithelial cells (arrow) were observed NF-κB/p65 positivity score: 0 (0–0.5)].

Examination under light microscopy of kidney tissues incubated with NF-κB/p65 primary antibodies revealed that the NF-κB/p65 positivity score of 0 (0–0) in the Control group increased to 3 (2–3) in the LPS group (Figure 4A,B; Table 6). In contrast, the NF-κB/p65 positivity score of 3 (2–3) in the LPS group decreased to 0 (0–1) in the PGPE100 group and 0 (0–0.5) in the PGPE300 (Figure 4C,D; Table 6).

### 3.5. Antibacterial Assay

#### 3.5.1. Agar Well Diffusion Method

**Figure 5:**
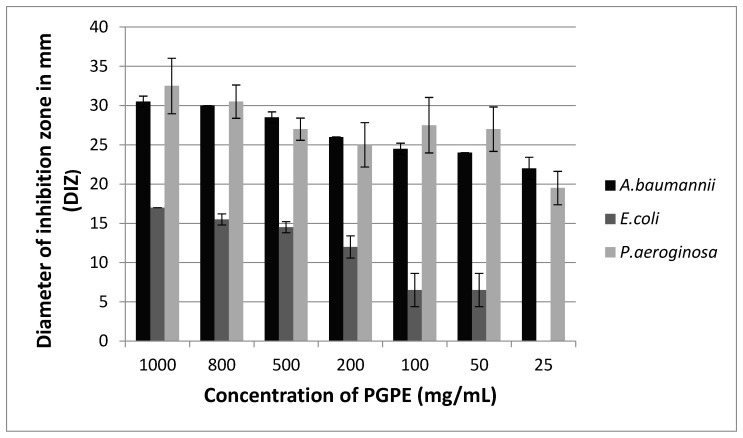
Inhibition zones of PGPE on *A. baumannii*, *E. coli*, and *P. aeruginosa*, respectively.

The antibacterial effect of PGPE in the form of inhibition zone diameter is given in Figure 5 and Table 7.

#### 3.5.2. MIC Assay

The MIC of PGPE was investigated against *A. baumannii*, *E. coli*, and *P. aeruginosa*. *A. baumannii* (8 µg/mL), *E. coli* (32 µg/mL), and *P. aeruginosa* (16 µg/mL) were not significantly different from the blank control, indicating the inhibition of bacterial growth. The results showed that the MIC values of *A. baumannii* were substantially lower than those of other bacteria (Table 7).

## 4. Discussion

The urinary tract is one of the primary infection sites where acute kidney injury (AKI) can grow in severe sepsis/septic shock [45]. Cohort studies defined that half of the patients developed sepsis in patients with AKI [46]. Septic AKI is a severe disease posing significant challenges to public health. Bouchard et al. reported that patients with septic AKI had greater severity of illness and higher mortality rates [47]. Rapid infection treatment, preventative measures against hospital-acquired infections and prophylactic antibiotic administration can reduce mortality and morbidity. Sepsis-specific treatment has proven mostly unsuccessful, as many microorganisms grow resistant to antimicrobials. According to the Centers for Disease Control and Prevention (CDC) 2019 AR Threats Report, the number of infections caused by 50% extended-spectrum beta-lactamase (ESBL)-producing *Enterobacteriaceae* has increased [48]. Carbapenem-resistant *Acinetobacter* and Carbapenem-resistant *Enterobacteriaceae* (CRE) are Gram-negative bacteria listed as urgent threats. ESBL-producing *Enterobacteriaceae* and multidrug-resistant *P. aeruginosa* are listed as serious threats [48]. The antimicrobial resistance problem has prompted researchers to investigate natural products to search for new drugs that could improve treatment outcomes. Traditional medicine practices have been widely used to treat diseases since ancient times. Due to their potent pharmacological properties, plants are used as a complementary treatment method.

*P. granatum* peel has well-known phytochemicals and has been approved as an excellent antimicrobial, anti-inflammatory, and antioxidant [18,19,22,23]. The antioxidant property of PGPE is derived from having a high content of polyphenols that scavenge free radicals and inhibit lipid oxidation [18,27]. In this study, several phenols were investigated, as given in Table 3. Punicalagine (PUN), epigallocatechin gallate (EGCG), chlorogenic acid (CGA), and ellagic acid (EA) were significantly higher than other compounds. The richest source was PUN, which was obtained with 50% MeOH. Guo et al. showed that pomegranate peel has the highest antioxidant activity compared to other fruit parts [25]. In addition, Yenil et al. showed that dry peel extract has more phenolic compounds than fresh peel extract, as excess water in the fresh peel negatively affects the yield. [26]. Cerda et al. reported that EA, GA (gallic acid), and PUN are the predominant phenolics of the fruit [24]. The phenolic compounds that PGPE highly contains scavenge free radicals and inhibit lipid oxidation in vitro [27].

The elevation of GSH levels serves as a protective mechanism against cellular damage. TBARS is a byproduct of lipid peroxidation, and LPS increases TBARS levels through oxidative stress [22,29]. In this study, the LPS group showed a significant increase in lipid peroxidation (TBARS) and GSH levels compared to the Control group. Results indicate that LPS induces oxidative stress, and GSH levels tend to increase the antioxidant response. PGPE treatment groups showed similar TBARS and GSH levels to the Control group, suggesting that these treatments might mitigate the effects of LPS. Many studies of the oxidative stress caused by sepsis have reported that TBARS levels are raised in tissues, and *P. granatum* supplements with high GSH levels eliminate the adverse situation by decreasing lipid peroxidation [49,50,51]. In Murthy et al.’s study, pretreatment of rats with PGPE reduced lipid peroxidation by 54% [52]. In addition, Shafik and co-workers conducted an analysis on rats. They emphasized that *P. granatum* remarkably improved liver injury through antioxidative and anti-inflammatory effects [53].

Phenolic compounds also possess anti-microbial properties against intestinal flora, particularly enteric pathogens [54,55,56]. They react with microbial cell membrane proteins or protein sulfhydryl groups, resulting in bacterial death due to membrane protein precipitation and the inhibition of enzymes [57]. Glycosyltransferase is an enzyme used in the biosynthesis of peptidoglycan [58]. The inhibition of glycosyltransferases leads to cell lysis, which is widely effective against *Staphylococcus aureus* and hemorrhagic *E. coli* [57]. In this study, inhibition zone diameters resulting from agar well diffusion analysis were evaluated according to the Clinical and Laboratory Standards Institute (CLSI) guidelines [42]. *A. baumannii* is classified as resistant (R) to CAZ, CIP, AMK, and MER. In contrast, PGPE (200 mg/mL) produced a 27 mm zone of inhibition, which is significantly larger than any of the routine antibiotics tested. *A. baumannii* may be classified as susceptible (S) to PGPE compared with the tested antibiotics. *E. coli* was found to be susceptible (S) to CAZ, CIP, AMK, and AMP. Although the inhibition zone diameter of PGPE was not directly compared with established CLSI guidelines, this zone size is close to that of AMP (21 mm), indicating that PGPE has moderate antibacterial activity against *E. coli* that may be classified as intermediate (I) to PGPE. *P. aeruginosa* found as susceptible (S) to CAZ, CIP, AMK, and TZP. The inhibition zone diameter of PGPE (20 mm) falls below the susceptible range for TZP (≥21 mm) and CIP (≥25 mm), but it is still at the upper limit of the intermediate (I) range. Although PGPE is less effective than CIP, CAZ, and AMK, it suggests moderate antibacterial activity against *E. coli* and *P. aeruginosa*, which is worth further investigation. In contrast, PGPE was highly effective against *A. baumannii*, potentially more so than the standard antibiotics used in this study. The results warrant further study to optimize the antibacterial potential of PGPE. Santos et al. have reported better/equally efficient than standard antibiotics on the bacterial strains, resulting in 9–38 mm inhibition zones. The peel extracts of *P. granatum* inhibited *Shigella, Salmonella* species, and *E. coli* to a considerable extent [55].

The minimum inhibitory concentration (MIC) method is used to determine the lowest concentration of an antimicrobial agent that inhibits the visible growth of a microorganism. It achieves quantitative data on the potency of the antimicrobial activity and gives a precise measurement of the concentration required to inhibit the growth of microorganisms. PGPE treatment considerably affected the Gram-negative activity, showing a dose-dependent effect. *A. baumannii*, *E. coli*, and *P. aeruginosa* gave measurable MICs (Table 7). A remarkable antibacterial effect of PGPE was found on *A. baumannii*. MIC value of *A. baumannii* is significantly lower than *E. coli* and *P. aeruginosa*. Based on CLSI guidelines, the MIC breakpoints of CAZ, CIP, AMK, and TZP against *P. aeruginosa*, PGPE showed a similar MIC to Piperacillin/Tazobactam (TZP), which is classified as sensitive at 16 µg/mL. The result suggests that PGPE has a potential antimicrobial activity at this concentration. PGPE exhibited limited antimicrobial activity against *E. coli*, showing resistance patterns similar to those of CAZ, CIP, AMK, and AMP breakpoints. On the contrary, PGPE demonstrated a certain level of effectiveness against *A. baumannii*. Given the susceptibility of CAZ and AMK, these antibiotics could be potential treatment options. However, the resistance to CIP and MER may impact treatment strategies.

The CRP level might reflect the in vivo inflammatory state in sepsis. Increased CRP levels can indicate the severity and intensity of inflammation. Zhou et al. compared sepsis models (cecal ligation and puncture and LPS), and identified elevated CRP levels as high sensitivity sepsis marker. CRP levels significantly increased at 24 h and 48 h post-sepsis induction [31]. Acute kidney injury is a rapid decrease in glomerular filtration rate that raises serum creatinine levels. The BUN and Cr levels reflect the renal function frequently used in the routine. In this study, the LPS group has experienced acute kidney injury, as evidenced by elevated BUN and Cr levels with high CRP levels. Data indicate impaired kidney function caused by sepsis. PGPE groups responded to a dose-dependent effect, particularly at high doses, which resulted in substantial improvements in both creatinine and BUN levels. Studies have shown that PGPE has a nephroprotective effect in sepsis and reduces renal function parameters as a consequence of the reduction in CRP levels [59,60].

Histopathologically, the absence of vacuolization, necrosis, and debris accumulation reflects the healthy and functional state of the kidneys. In the Control group, normal renal corpuscles, glomeruli, and proximal and distal tubules were observed, which means renal tissue structure was preserved. On the other hand, the LPS group displayed severe histopathological changes, including widespread vacuolization in the cytoplasm of renal corpuscles and tubular epithelial cells. In addition, a striking loss of brush border structures in proximal tubular epithelial cells suggests a crucial role in absorption and indicates significant functional impairment. Findings suggest that LPS triggers intense inflammatory responses and oxidative stress in renal tissues. The PGPE100 group demonstrated a significant reduction in vacuolization in both the proximal and distal tubules, suggesting a partial restoration of cellular damage. Although necrotic epithelial cells and debris accumulation were still present, they were less prominent than in the LPS group, indicating that PGPE100 partially protected against LPS-induced damage. Notably, the brush border structures in the proximal tubules appeared intact, signifying that PGPE100 preserved some functional aspects of the tubular epithelium despite the injury. The higher dose of PGPE (PGPE300) provided more vital protection that meant that debris accumulation in the tubular lumens was minimal, and the proximal tubule epithelial cells exhibited typical brush border structures, highlighting the near-complete preservation of renal structure and function.

The main reason for sepsis-associated damage is the triggering and intense amplification of inflammatory cytokines. Increased free oxygen radicals (ROS) resulting from oxidative stress caused by cellular damage trigger NF-κB-mediated inflammation [47]. TLR4 is a receptor that is best known for recognizing LPS and initiates an immune signaling cascade [32]. This cascade leads to the activation of NF-κB which is a transcription factor that regulates the expression of inflammatory genes. LPS exposure significantly upregulates both TLR4 and NF-κB/p65 positivity, confirming their involvement in the inflammatory damage. In this study, the Control group indicated minimal expression of TLR4 in normal kidney tissue, which aligns with its low baseline activity. In the LPS group, elevated TLR4 expression suggests an activated inflammatory response in the kidney tissue due to lipopolysaccharide exposure. In the PGPE100 and PGPE300 groups, decreased TLR4 levels suggest that PGPE treatment modulates TLR4 signaling, likely reducing the activation of the inflammatory cascade. The fact that the TLR4 score returned to near-control levels in both treatment groups implies that PGPE suppresses TLR4-mediated inflammatory responses in a dose-dependent manner. In addition, there was a robust activation of NF-κB determined in the LPS group. TLR4 signaling leads to NF-κB activation, which then induces the expression of pro-inflammatory cytokines. PGPE treatment strongly inhibits NF-κB activation, likely by preventing the signaling cascade initiated by TLR4. In particular, the near-complete suppression of NF-κB activity in the PGPE300 group suggests a dose-dependent anti-inflammatory effect of *P. granatum* peel extract, as it limits the transcription of pro-inflammatory genes. Previous studies have shown TLR4 levels and NF-κB activation in S-AKI [28,45,47]. The precipitation of oxidative stress leads to an increase in proinflammatory cytokine production. Renal injury is inevitable if the inflammation is not brought under control. Active compounds of *P. granatum* enhanced the adipokine pathway and reduced oxidative stress and downregulated nuclear factor κB activation and phosphodiesterase 4 expressions [24]. Similarly, to the present study, many studies showed increased TLR4/NF-κB levels in S-AKI [16,37].

## 5. Conclusions

This study employed biochemical and histopathological means to show that PGPE can reduce sepsis-associated kidney injury, which is difficult to treat because of antibiotic resistance. The findings showed that PGPE prevents S-AKI by exhibiting antioxidant and anti-inflammatory effects.

## Figures and Tables

**Table 1:** Tubular necrosis scoring modified from Sung et al. [40].

Observations	Score				
Deterioration of brush border structure in proximal tubules (%)	**0**	**1**	**2**	**3**	**4**
	none	≤10%	10–25%	26–75%	≥75%
Debris accumulation in the lumen (%)	none	≤10%	10–25%	26–75%	≥75%
Loss of tubular epithelial cells connections (%)	none	≤10%	10–25%	26–75%	≥75%

**Table 2:** Kidney Histopathological Damage Score (KHDS).

Observations	Score			
TNS	**0**	**1**	**2**	**3**
	none	≤10%	10–25%	26–75%
Hemorrhage	none	≤10%	10–25%	26–75%
Atypical Renal Corpuscle	none	≤10%	10–25%	26–75%

**Table 3:** Phenolic Compounds of *P. granatum* Peel (µg/g).

Compounds	% MeOH		
	50	80	100
Ellagic acid (EA)	46.385	70.971	42.306
Gallic acid (GA)	2.894	1.804	1.519
Ferulic acid (FA)	4.427	4.075	1.726
Quercetin (Q)	0.789	0.818	0.812
*p*-coumaric acid (*p*-CA)	0.192	0.175	0.101
Caffeic acid (CA)	1.586	1.681	0.773
2,4-dihydroxybenzoic acid (2,4-DHBA)	11.193	11.538	9.934
Epigallocatechin gallate (EGCG)	186.88	274.901	191.394
Catechin hydrate (CH)	5.674	5.488	5.399
Caffeine (CAF)	16.328	21.708	7.547
Chlorogenic acid (CGA)	60.394	28.480	21.575
Ursolic acid (UA)	2.44	3.380	10.719
Punicalagin (A) (PUN)	4528.030	1451.663	1345.471
Punicalagin (B) (PUN)	2591.466	827.183	639.108

**Table 4:** Biochemical Analysis Results (mean ± Standard deviation).

Groups	TBARS (nmol/g Tissue)	GSH (µmol/g Tissue)
Control	115 ± 13	7.93 ± 0.85
LPS	223 ± 51 ^b^	10.63 ± 2.47 ^a^
PGPE100	147 ± 35	8.70 ± 1.04
PGPE300	146 ± 17	8.85 ± 1.69

^a^ *p* < 0.05: vs. the Control group, ^b^ *p* ≤ 0.001: vs. the LPS group. One-Way ANOVA/Tukey HSD.

**Table 5:** Tubular Necrosis Score (TNS) Results (Median (25–75% interquartile range)).

Groups	Brush Border Damage Score	Luminal Debris Accumulation Score	Loss of Tubular Epithelial Cells Score	TNS
Control	0 (0–0.5)	0 (0–0)	0 (0–0)	0 (0–0.5)
LPS	2.5 (2–3) ^a^	2 (1–2) ^a^	3 (2–3) ^a^	7.5 (5–8) ^a^
PGPE100	1 (0–1) ^b^	0 (0–0.5) ^b^	0.5 (0–0.5) ^b^	1.5 (0.5–2) ^a,b^
PGPE300	0.5 (0–0.5) ^b^	0 (0–0.5) ^b^	0 (0–0.5) ^b^	1 (0.5–1) ^a,b^

^a^ *p* = 0.001 versus to Control group, ^b^ *p* = 0.001 versus to LPS group, Kruskal Wallis/Dunn test.

**Table 6:** IHC positivity grading results (Median (25–75% interquartile range)).

Group	TLR4 Positivity Scores	NF-κB/p65 Positivity Scores
Control	0 (0–0.5)	0 (0–0)
LPS	1.5 (1–2) ^a^	3 (2–3) ^a^
PGPE100	0.5 (0–0.5) ^b^	0 (0–1) ^b^
PGPE300	0.5 (0–0.5) ^b^	0 (0–0.5) ^b^

^a^ *p* = 0.001 versus to the Control group, ^b^ *p* = 0.001 versus to the LPS group, Kruskal Wallis/Dunn test.

**Table 7:** Agar well diffusion method and MIC values, comparative inhibition zone diameters of PGPE with antibiotics.

Bacteria	Zone Diameter (mm)			MIC (µg/mL)	Appearance in Plate
	Antibiotics		PGPE (200 mg/mL)		
*A. baumannii*	CAZ	-	27	8	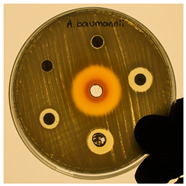
	CIP	-			
	AMK	12			
	MER	10			
*E. coli*	CAZ	30	21	32	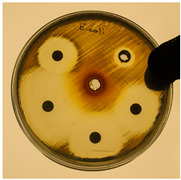
	CIP	43			
	AMK	27			
	AMP	21			
*P. aeruginosa*	CAZ	26	20	16	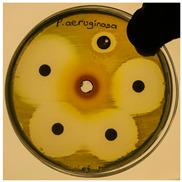
	CIP	40			
	AMK	30			
	TZP	28			

AMK: Amikacin, AMP: Ampicillin, CAZ: Ceftazidime, CIP: Ciprofloxacin, MER: Meropenem TZP: Piperacillin/Tazobactam PGPE: *P. granatum* Peel Extract. -: No inhibition. Zone diameter and MIC breakpoints were evaluated per CLSI.

## Data Availability

Original data supporting the findings of this study are available. No copyright permissions are required for the figures in this study.
